# Tumor budding is a significant indicator of a poor prognosis in lung squamous cell carcinoma patients

**DOI:** 10.3892/mmr.2012.1048

**Published:** 2012-08-27

**Authors:** RYOTA MASUDA, HIROSHI KIJIMA, NAOKO IMAMURA, NAOHIRO ARUGA, YUSUKE NAKAMURA, DAISUKE MASUDA, HARUKA TAKEICHI, NOBUSUKE KATO, TOMOKI NAKAGAWA, MAKIKO TANAKA, SADAKI INOKUCHI, MASAYUKI IWAZAKI

**Affiliations:** 1 Department of General Thoracic Surgery, Tokai University School of Medicine, Isehara, Kanagawa 259-1193; 2 Department of Pathology and Bioscience, Hirosaki University Graduate School of Medicine, Hirosaki, Aomori 036-8562; 3 Department of Critical Care and Emergency Medicine, Tokai University School of Medicine, Isehara, Kanagawa 259-1193, Japan

**Keywords:** lung cancer, squamous cell carcinoma, patient prognosis, tumor budding

## Abstract

Lung cancer is a leading cause of cancer mortality worldwide and patients occasionally develop local recurrence or distant metastasis soon after curative resection. Reports of new therapeutic strategies for lung squamous cell carcinoma (SqCC) are extremely rare, while selective anticancer therapy has been reported for lung adenocarcinoma. The aim of this study was to identify clinicopathological prognostic factors for SqCC. We analyzed tumor budding and infiltrative patterns (INF) in 103 cases of surgically-resected SqCC. Tumor infiltrative patterns were classified into three groups (INFa, b and c) and INFc was infiltrative growth at the tumor invasive front. The cases with an INFc component [INFc(+)]were significantly associated with venous invasion (P=0.014) and the scirrhous stromal type (P<0.001). The overall survival rate of patients with INFc(+) was significantly lower than that of patients without the INFc component [INFc(−); P=0.003]. Tumor budding was defined as a single cancer cell or a small nest of up to four cancer cells within stromal tissue. The cases with tumor budding [Bud(+)] were significantly associated with lymph node metastasis (P=0.001), lymphatic invasion (P=0.002), INFc(+) (P<0.001) and the scirrhous stromal type (P=0.014). Patients with the Bud(+) type had a lower overall survival rate than patients with the Bud(−) type (P<0.001). Multivariate analysis demonstrated that tumor budding [hazard ratio (HR), 2.766; 95% confidence interval (CI), 1.497–5.109] and lymph node metastasis (HR, 1.937; 95% CI, 1.097–3.419) were independent predictors of mortality. In conclusion, tumor budding is a significant indicator of a high malignant potential and poor prognosis in SqCC of the lung.

## Introduction

Lung cancer is the most common type of cancer and the leading cause of cancer mortality worldwide (1). Despite complete surgical resection, the prognosis of lung cancer is generally poor (2), with recurrence rates of 15–30% and 5-year survival rates of 60–70% (3). Lung cancer is commonly classified into four types: squamous cell carcinoma (SqCC), adenocarcinoma, large cell carcinoma and small cell carcinoma, based on the histological features (2,4,5). Patient prognosis with SqCC is more favorable than the other histological types (2,6). Customized chemotherapy for unresectable or recurrent lung cancers is more frequently used for adenocarcinoma than for SqCC (7,8). In addition, molecular targeting therapies, including bevacizumab (9,10), erlotinib (11,7) and gefitinib (7) have been developed recently. By contrast, there are few therapeutic options for recurrent SqCC. Therefore, it is necessary to examine the histopathological features to clarify a poor prognosis group for SqCC.

Invasive patterns have been considered as prognostic factors for other solid cancers (12–14). Tumor budding is believed to be a significant invasive pattern and has attracted interest, and is defined as isolated single cancer cells or a cluster of cancer cells composed of fewer than five cells (15,16). Tumor budding has been reported to be a prognostic factor not only in the gastrointestinal tract (16–18), but also in the tongue (19) and larynx (20). The gastrointestinal pathology commonly describes the budding grade at the invasive front of the cancer. However, evaluation of the budding grade is believed to be difficult at the invasive front of lung SqCC, but tumor budding was observed in the fibrosis and collapse at the tumor-stroma interface of lung adenocarcinoma (21).

The aim of the present study was to identify indicators that may be used to predict a poor prognosis for patients with SqCC based on tumor budding and other clinicopathological factors.

## Materials and methods

### Lung cancer specimens

The cancer tissue specimens were obtained from surgically resected lung SqCC cases following the receipt of patient informed consent, according to the Institutional Review Board (IRB) of Tokai University Hospital. The 103 patients (97 males and 6 females; age range, 43–85 years; mean age, 67.2±9.1 years) with lung SqCC underwent radical surgery (lobectomy and mediastinal lymphadenectomy) at Tokai University Hospital (Kanagawa, Japan). The tumor stages were defined according to the TNM classification of the International Union Against Cancer (UICC) (22) and the histological types were defined according to the World Health Organization classifications (6). The median postoperative follow-up duration was 1,528 (41–3,837) days.

### Histological examination

The lung tissue specimens for histological analysis were fixed with 10% buffered formalin for 24–48 h and routinely embedded in paraffin. The tumors were cut at 5–10-mm intervals. Tumor and lymphatic invasion were examined on 4-μm thick sections stained with hematoxylin and eosin. Vascular and pleural invasion were evaluated using the Verhoeff-van Gieson method.

**Figure 1:**
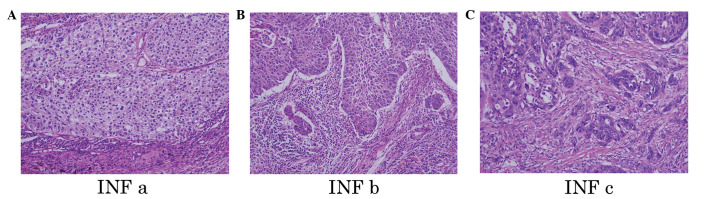
Microscopic findings of lung squamous cell carcinoma (hematoxylin and eosin staining). Tumor infiltrative patterns (INF) at the invasive front are classified into three groups: (A) INFa, cancer nests show an expansion in growth and a distinct border with the surrounding tissue; (B) INFb, the manner of growth and invasive pattern are between those of INFa and INFc; and (C) INFc, cancer nests show infiltrative growth and the borderline with the surrounding tissue is unclear.

Tumor infiltrative patterns (INF) at the invasive front were classified into three groups according to the general criteria for gastric cancer studies (23–25): INFa, cancer nests demonstrate an expansion of growth and a distinct border with the surrounding tissue; INFb, the manner of growth and invasive pattern are between those of INFa and INFc; and INFc, cancer nests show infiltrative growth and the borderline with the surrounding tissue is unclear (Fig. 1). The stromal types, i.e. cancer-stroma relationship patterns, were also classified into three groups: medullary type, stroma is limited; intermediate type, quantity of stroma is intermediate between those of the scirrhous and medullary types; and scirrhous type, stroma is abundant (23).

**Figure 2:**
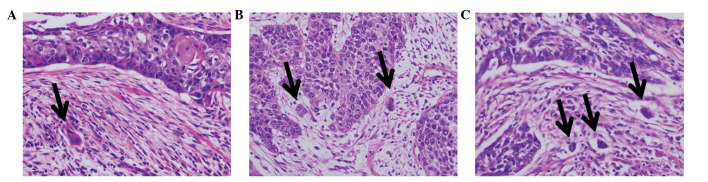
Microscopic findings of lung squamous cell carcinoma (hematoxylin and eosin staining). Tumor budding is defined as single cancer cells and/or clusters composed of up to four cancer cells. (A) One, (B) two and (C) three budding foci.

Tumor budding was defined as single cancer cells and clusters composed of up to four cancer cells (26). These cancer cells were observed in cancer-stroma lesions at the invasive front of the tumor. Numbers of tumor budding foci were counted in the histological fields in which the tumor budding intensity was maximal within the histological section, using a ×20 objective lens as described previously (16). In the present study, the cases were classified into two groups: tumor budding-positive/negative [Bud(+)/Bud(−)]. According to the number of tumor buds per field, the cases were subclassified into three groups: grade 0, no budding foci; grade 1, up to two budding foci; and grade 2, three or more budding foci (Fig. 2).

### Statistical analysis

Univariate analyses (Chi-square tests) were primarily used for selecting variables on the basis of a value of P<0.05. A Cox proportional hazards regression analysis was used to determine the net effect of each predictor while controlling for the effects of the other factors by univariate and multivariate analysis. Hazard ratios (HR) and their 95% confidence intervals (CI) were used to assess the independent contributions of significant factors. P<0.05 was considered to indicate a statistically significant result.

The patient survival time was measured between the date of surgery and mortality from all causes (without discrimination between mortalities resulting from lung carcinoma and other causes). Survival curves were created using the Kaplan-Meier method and compared using the log-rank test. All analyses were performed using the SPSS II statistical software package (version 19.0; SPSS Inc., Tokyo, Japan).

## Results

### Tumor INF of lung SqCC

**Figure 3:**
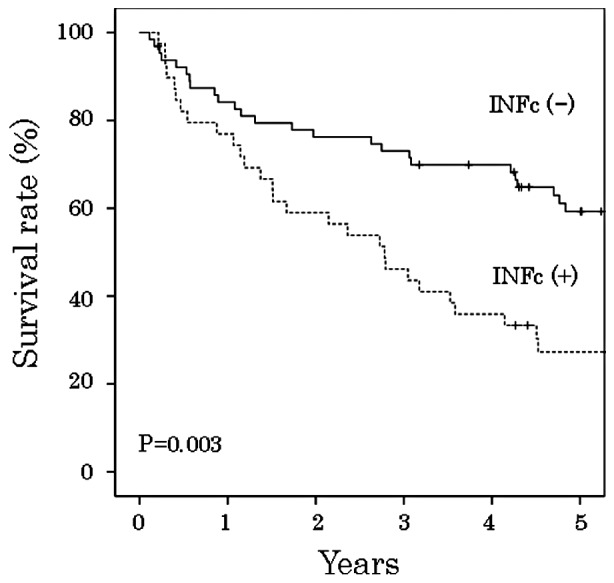
Tumor infiltrative patterns and cumulative survival of patients with lung squamous cell carcinoma. INFc(−)/INFc(+), tumor infiltrative pattern group c component-negative/positive.

Tumor INF were classified into three groups, INFa, b and c, but certain cases had two components of INF, including INFa>b. The numbers of these INF groups were as follows: INFa (11, 10.7%); INFa>b (10, 9.7%); INFb (43, 41.7%); INFb>c (31, 30.1%); INFb<c (4, 3.9%); and INFc (4, 3.9%). The cases with an INFc component [INF(+) = INFb>c, INFb<c and INFc] showed a poor outcome, compared with those without the INFc component [INFc(−) = INFa, INFa>b and INFb] (Table I). The stromal types, i.e., the cancer-stroma relationship patterns, were divided into three groups, as follows: medullary (39, 37.9%); intermediate (31, 30.1%); and scirrhous types (33, 32.0%). The correlations between the tumor INF and clinicopathological features are summarized in Table II. INFc(+) cases showed significantly higher incidences of venous invasion (P=0.014) and the scirrhous stromal type (P<0.001) than INFc(−) cases. Using the Kaplan-Meier method and log-rank test, the overall patient survival rate following curative resection was lower in INFc(+) than in INFc(−) cases (P=0.003; Fig. 3).

### Tumor budding of lung SqCC

**Figure 4:**
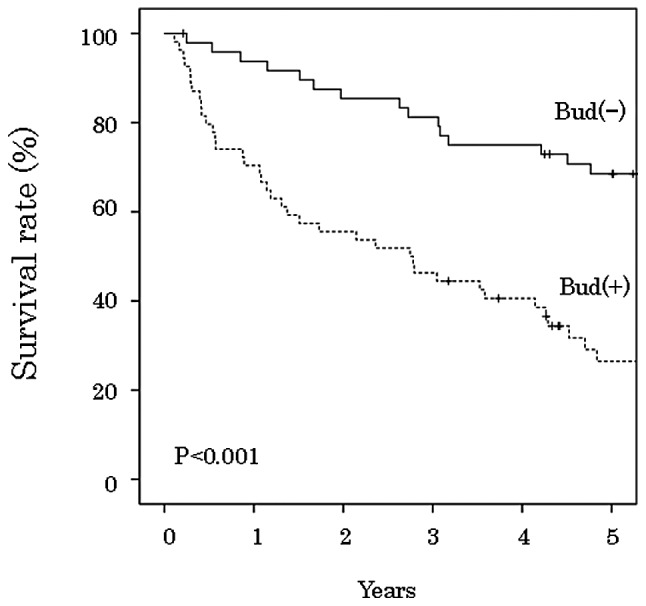
Tumor budding and cumulative survival of patients with lung squamous cell carcinoma.

Regarding tumor budding, there were 54 (52.4%) cases with the Bud(+) type and 49 (47.6%) cases with the Bud(−) type. Bud(+) cases included 22 cases (40.8%) with one budding focus, 20 cases (37.0%) with two budding foci, 6 cases (11.1%) with three budding foci and 6 cases (11.1%) with >four budding foci. The correlations between the tumor budding types and clinicopathological features are summarized in Table III. Lymph node metastasis (P=0.001), lymphatic invasion (P=0.002), the scirrhous stromal type (P=0.016) and infiltrative pattern (P<0.001) showed significantly higher incidences in the Bud(+) type. The overall survival rate following curative resection was lower in patients with the Bud(+) type than in those with the Bud(−) type (P<0.001, log-rank test, Fig. 4). Using the Kaplan-Meier method and log-rank test, the patient outcome of the Bud(−) group was significantly better than that of the Bud(+) group; (P<0.001; Fig. 4).

### Clinicopathological significance of tumor INF/tumor budding

The univariate analyses identified six factors associated with increased mortality in patients with lung SqCC (Table IV): tumor size (HR, 1.897; 95% CI, 1.059–3.396); lymph node metastasis (HR, 3.028; 95% CI, 1.785–5.136); lymphatic invasion (HR, 3.298; 95% CI, 1.827–5.952); histological differentiation (HR, 2.092; 95% CI, 1.050–4.168); tumor infiltrative patterns (HR, 2.209; 95% CI, 1.301–3.749); and tumor budding (HR, 3.276; 95% CI, 1.841–5.827). The scirrhous stromal type did not significantly affect the survival of patients with lung SqCC (HR, 1.229; 95% CI, 0.706–2.139). The multivariate analysis is summarized in Table V. Tumor budding (HR, 2.766; 95% CI, 1.497–5.109) and lymph node metastasis (HR, 1.937; 95% CI, 1.097–3.419) remained significant predictors of patient mortality.

## Discussion

In the present study, we analyzed tumor budding and the other clinicopathological factors of lung SqCC and clarified that tumor budding and the INFc(+) type are correlated with the malignant potential of lung SqCC. Generally, 52% of the SqCC cases had foci of tumor budding, i.e., the Bud(+) type, and showed higher rates of lymph node metastasis and a lower overall survival rate. A previous study reported that tumors with a single-cell invasive component were a useful prognostic factor for small peripheral SqCC of the lung, while there was no significant association between patterns and patient prognosis (27). To the best of our knowledge, the present study is the first to describe the correlation between tumor budding and the prognosis of patients with SqCC of the lung.

Evaluation of the budding grades varies in different histological types, while it has been strictly defined based on histological criteria. Frequent budding foci tend to be observed in adenocarcinoma. In numerous organs, ten budding foci of adenocarcinoma are set as a cut-off value for high-grade budding (16,21). By contrast, five budding foci per high-power field are set as the cut-off value for SqCC (17,19,20). In this study, we used the actual numbers of budding foci for histological evaluation since only a few budding foci were found in the lung SqCC samples. Patient prognoses were significantly different between the cases with and without budding foci, i.e., all or no tumor budding in lung SqCC, while there was no significant difference among the numbers of budding foci. Therefore, the presence of budding foci is an important indicator directly associated with the prognosis of lung SqCC patients.

The conventional INF factors are classified according to the infiltration patterns and show the entire growth patterns at the tumor invasive front. However, INF factors do not extensively demonstrate actual aggressive tumor growth patterns. In the present study, a close correlation was observed between Bud(+) and INFc(+) (P<0.001). Bud(+) and INFc(+) are differently defined, while the two factors reflect local INF of tumor growth. Therefore, multivariate analysis revealed tumor budding, but not INF, as a significant indicator of high malignant potential and a poor patient prognosis.

Several studies have reported several transient molecular alterations which occur during tumor budding (16,17,19,21,28) and experimental analyses have demonstrated interactions between cellular adhesion molecules, including β-catenin, E-cadherin, CD44 and laminin-5γ2, and tumor budding in colorectal carcinoma (29,30). Previously, other studies reported that the overexpression of laminin-5γ2 is a significant prognostic factor in lung adenocarcinoma (21,30). However, there have been no immunohistochemical/molecular examinations using lung SqCC cases. Therefore, we plan to perform studies to clarify the pathological mechanisms of budding formation in lung SqCC using immunohistochemical/molecular analyses in the future.

In conclusion, tumor budding, i.e., the Bud(+) type, of lung SqCC shows locally aggressive growth and is a useful indicator of the lymph node status and prognosis.

## Figures and Tables

**Table I:** Infiltration patterns in lung squamous cell carcinoma patients.

Variable	No. of patients (%)	P-value	Hazard ratio	95% confidence interval
INFa	11 (10.7)	0.134	1.203	0.945–1.531
INFa>b, b, b>c, b<c, c	92 (89.3)			
INFa, a>b	21 (20.4)	0.859	1.062	0.547–2.060
INFb, b>c, b<c, c	82 (79.6)			
INFa, a>b, b	64 (62.1)	0.003	2.209	1.301–3.749
INFb>c, b<c, c	39 (37.9)			
INFa, a>b, b, b>c	95 (92.2)	0.363	1.535	0.610–3.860
INFb<c, c	8 (7.8)			
INFa, a>b, b, b>c, b<c	99 (96.1)	0.961	1.036	0.252–4.255
INFc	4 (3.9)			

INF, tumor infiltative pattern; INFa, cancer nests demonstrate an expansion of growth and a distinct border with the surrounding tissue; INFb, the manner of growth and invasive pattern are between those of INFa and INFc; and INFc, cancer nests show infiltrative growth and the borderline with the surrounding tissue is unclear.

**Table II:** Tumor infiltration patterns and clinicopathological features of lung squamous cell carcinoma.

Variable	No. of patients (%)	INFc(−) (%)	INFc(+) (%)	P-value
Age at surgery (years)				
<68	53 (51.5)	37 (69.8)	16 (30.2)	0.098
≥68	50 (48.5)	27 (54.0)	23 (46.0)	
Gender				
Male	97 (94.2)	61 (62.9)	36 (37.1)	0.528
Female	6 (5.8)	3 (50.0)	3 (50.0)	
Tumor size (mm)				
≤30	39 (37.9)	23 (59.0)	16 (41.0)	0.606
>30	64 (62.1)	41 (64.1)	23 (35.9)	
Lymph node metastasis				
n(−)	70 (68.0)	46 (65.7)	24 (34.3)	0.276
n(+)	33 (32.0)	18 (54.5)	15 (45.5)	
Lymphatic invasion				
ly(0, 1)	84 (81.6)	55 (65.5)	29 (34.5)	0.142
ly(2, 3)	19 (18.4)	9 (47.4)	10 (52.6)	
Venous invasion				
v(−)	53 (51.5)	39 (73.6)	14 (26.4)	0.014
v(+)	50 (48.5)	25 (50.0)	25 (50.0)	
Histological differentiation				
Well, moderate	90 (87.4)	53 (58.9)	37 (41.1)	0.074
Poor	13 (12.6)	11 (84.6)	2 (15.4)	
Stromal type				
Medullary, intermediate	70 (68.0)	53 (75.7)	17 (24.3)	<0.001
Scirrhous	33 (32.0)	11 (33.3)	22 (66.7)	

n(−)/n(+), lymph node metastasis-negative/positive; v(−)/v(+), venous invasion-negative/positive; INFc(−)/INFc(+), tumor infiltative pattern group c component-negative/positive.

**Table III:** Tumor budding and clinicopathological features of lung squamous cell carcinoma.

Variable	No. of patients (%)	Bud(−) (%)	Bud(+) (%)	P-value
Age at surgery (years)				
<68	53 (51.5)	27 (50.9)	26 (49.1)	0.481
≥68	50 (48.5)	22 (44.0)	28 (56.0)	
Gender				
Male	97 (94.2)	44 (45.4)	53 (54.6)	0.071
Female	6 (5.8)	5 (83.3)	1 (16.7)	
Tumor size (mm)				
≤30	39 (37.9)	19 (48.7)	20 (51.3)	0.856
>30	64 (62.1)	30 (46.9)	34 (53.1)	
Lymph node metastasis				
n(−)	70 (68.0)	41 (58.6)	29 (41.4)	0.001
n(+)	33 (32.0)	8 (24.2)	25 (75.8)	
Lymphatic invasion				
ly(0, 1)	84 (81.6)	46 (54.8)	38 (45.2)	0.002
ly(2, 3)	19 (18.4)	3 (15.8)	16 (84.2)	
Venous invasion				
v(−)	53 (51.5)	27 (50.9)	26 (49.1)	0.481
v(+)	50 (48.5)	22 (44.0)	28 (56.0)	
Histological differentiation				
Well, moderate	90 (87.4)	43 (47.8)	47 (52.2)	0.913
Poor	13 (12.6)	6 (46.2)	7 (53.8)	
Stromal type				
Medullary, intermediate	70 (68.0)	39 (55.7)	31 (44.3)	0.016
Scirrhous	33 (32.0)	10 (30.3)	23 (69.7)	
Infiltrating pattern				
INFc(−)	64 (62.1)	41 (64.1)	23 (35.9)	<0.001
INFc(+)	39 (37.9)	8 (20.5)	31 (79.5)	

n(−)/n(+), lymph node metastasis-negative/positive; v(−)/v(+), venous invasion-negative/positive; INFc(−)/INFc(+), tumor infiltrative pattern group c component-negative/positive; Bud(−)/Bud(+), budding-negative/positive.

**Table IV:** Clinicopathological features and survival in lung squamous cell carcinoma patients.

Variable	No. of patients (%)	P-value	Hazard ratio	95% confidence interval
Age at surgery (years)				
<68	53 (51.5)	0.131	1.502	0.885–2.548
≥68	50 (48.5)			
Gender				
Male	97 (94.2)	0.904	0.939	0.339–2.602
Female	6 (5.8)			
Tumor size (mm)				
≤30	39 (37.9)	0.031	1.897	1.059–3.396
>30	64 (62.1)			
Lymph node metastasis				
n(−)	70 (68.0)	<0.001	3.028	1.785–5.136
n(+)	33 (32.0)			
Lymphatic invasion				
ly(0, 1)	84 (81.6)	<0.001	3.298	1.827–5.952
ly(2, 3)	19 (18.4)			
Venous invasion				
v(−)	53 (51.5)	0.145	1.486	0.873–2.530
v(+)	50 (48.5)			
Histological differentiation				
Well, moderate	90 (87.4)	0.036	2.092	1.050–4.168
Poor	13 (12.6)			
Stromal type				
Medullary, intermediate	70 (68.0)	0.465	1.229	0.706–2.139
Scirrhous	33 (32.0)			
Infiltrating pattern				
INFc(−)	64 (62.1)	0.003	2.209	1.301–3.749
INFc(+)	39 (37.9)			
Budding				
Bud(−)	49 (47.6)	<0.001	3.276	1.841–5.827
Bud(+)	54 (52.4)			

n(−)/n(+), lymph node metastasis-negative/positive; INFc(−)/INFc(+), INFc component-negative/positive; Bud(−)/Bud(+), budding-negative/positive.

**Table V:** Multivariate analysis of clinicopathological features and survival of lung squamous cell carcinoma patients.

Variable	No. of patients (%)	P-value	Hazard ratio	95% confidence interval
Tumor size (mm)				
≤30	39 (37.9)	0.064	1.774	0.968–3.250
>30	64 (62.1)			
Lymph node metastasis				
n(−)	70 (68.0)	0.023	1.937	1.097–3.419
n(+)	33 (32.0)			
Budding				
Bud(−)	49 (47.6)	0.001	2.766	1.497–5.109
Bud(+)	54 (52.4)			

n(−)/n(+), lymph node metastasis-negative/positive; Bud(−)/Bud(+), budding-negative/positive.
